# Immunotherapy for glioblastoma: the promise of combination strategies

**DOI:** 10.1186/s13046-022-02251-2

**Published:** 2022-01-25

**Authors:** Mathilde Bausart, Véronique Préat, Alessio Malfanti

**Affiliations:** UCLouvain, Louvain Drug Research Institute, Advanced Drug Delivery and Biomaterials, Avenue Mounier 73 B1.73.12, 1200 Brussels, Belgium

**Keywords:** Brain cancer, Glioblastoma, Combination immunotherapy, Immune checkpoint blockade, Cancer vaccine

## Abstract

Glioblastoma (GBM) treatment has remained almost unchanged for more than 20 years. The current standard of care involves surgical resection (if possible) followed by concomitant radiotherapy and chemotherapy. In recent years, immunotherapy strategies have revolutionized the treatment of many cancers, increasing the hope for GBM therapy. However, mostly due to the high, multifactorial immunosuppression occurring in the microenvironment, the poor knowledge of the neuroimmune system and the presence of the blood−brain barrier, the efficacy of immunotherapy in GBM is still low. Recently, new strategies for GBM treatments have employed immunotherapy combinations and have provided encouraging results in both preclinical and clinical studies. The lessons learned from clinical trials highlight the importance of tackling different arms of immunity. In this review, we aim to summarize the preclinical evidence regarding combination immunotherapy in terms of immune and survival benefits for GBM management. The outcomes of recent studies assessing the combination of different classes of immunotherapeutic agents (e.g., immune checkpoint blockade and vaccines) will be discussed. Finally, future strategies to ameliorate the efficacy of immunotherapy and facilitate clinical translation will be provided to address the unmet medical needs of GBM.

## Background

Glioblastoma (GBM) is the most common and aggressive malignant tumor of the central nervous system (CNS) [1, 2]. GBM is a grade IV diffuse astrocytoma that is thought to arise from neural stem cells or progenitor cells, such as oligodendrocyte precursor cells [2–4]. Approximately 90% of GBM cases are considered primary GBM, with fast and de novo expansion and without any sign of less malignant precursor tumors. Primary GBM often develops in elderly patients and shows a much poorer prognosis than secondary GBM, which originates from grade II and III astrocytomas, oligodendrogliomas or oligoastrocytomas and most likely manifesting in younger patients [3, 5].

Standard of care (SOC) therapy aims at increasing patient life expectancy and focuses on maximal and safe surgical resection combined with radiotherapy (RT) and adjuvant chemotherapy in the form of oral delivery of temozolomide (TMZ) [6]. Despite this treatment, the median survival of patients diagnosed with GBM is approximately 15 months, with a 2-year life expectancy of less than 30% [7]. For patients with unresectable GBM (up to 35–40% of patients), the prognosis is even poorer [8–10]. Indeed, microscopic infiltration of GBM cells and the tumor location render total resection difficult or even impossible and produce inevitable recurrences [9]. Finding new therapies for GBM is therefore an urgent unmet need, although it is very challenging because of unique characteristics of GBM and its tumor microenvironment (TME). GBM are characterized by intratumoral and intertumoral heterogeneity, highly invasive and infiltrative cell properties and an immunosuppressive TME promoting GBM growth via complex interactions [11].

Immunotherapies, by re-educating and harnessing the patient’s immune response against tumors, hold great promise for cancer treatment. These methods have become increasingly used in the treatment of different kinds of cancers, including brain cancers [12, 13]. Current immunotherapy strategies used to treat cancers are mainly based on immune checkpoint blockade (ICB) agents [14, 15], but also therapeutic vaccines [16, 17], adoptive cell therapy [18, 19], monoclonal antibodies (mAbs) [20] and oncolytic viruses [21].

Treatment with ICBs has shown remarkable success in a population of patients with melanomas and other tumors [22–25]. Therapeutic vaccines have also emerged as promising cancer treatments, with currently 3 therapeutic cancer vaccines approved by the Food and Drug Administration (FDA) [26]. However, even though immunotherapies have shown survival benefit for some proportions of patients with solid tumors, most patients still do not respond to immunotherapy. Less than 15% of cancer patients currently respond to ICBs [27]. Furthermore, these strategies are not as effective as would be desired for GBM treatment. To date, phase III clinical studies with ICB and vaccine therapies have shown no major benefit of immune modulation for GBM treatment or patient survival [13, 28].

A recent clinical study, however, demonstrated that administration of anti-programmed cell death protein 1 (PD-1) mAbs prior to tumor resection increased local and systemic antitumor immune responses [29]. Additionally, interim results of a phase II clinical study evaluating the combination of an allogeneic/autologous therapeutic GBM vaccine in combination with granulocyte-macrophage colony-stimulating factor (GM-CSF), cyclophosphamide and bevacizumab demonstrated a significant survival benefit [30]. These results raise hope for research on GBM immunotherapy treatments. Immune modulation in combination with other treatments has shown encouraging preclinical results. This review summarizes some of these promising combination strategies for the treatment of GBM. We will particularly focus on combinations including ICBs, as they are the most studied combination strategies including immunotherapy for GBM.

## The BBB and immune microenvironment in GBM: implications for the development of new therapies

To ensure proper neuronal function, the brain has to be maintained in a homeostatic state. This implies regulation of the influx/efflux of cells, molecules and ions [31]. Two major barriers contribute to separating the CNS from the variable environment of blood: (i) the blood−cerebrospinal fluid barrier, formed by the choroid plexus epithelium and separating the cerebrospinal fluid from the blood; and (ii) the blood−brain barrier (BBB), formed by endothelial cells of the capillary of brain parenchyma and separating the blood from the brain interstitial fluid [32, 33].

Due to its anatomical structure and vascular organization, the BBB is the most selective barrier [34]. For this reason, the BBB is also an important obstacle for the development of successful GBM treatments. While it has been shown to be disrupted in GBM, an intact BBB is still found peritumorally [35, 36]. This heterogeneous disruption leads to protection of most infiltrative components of GBM and limits the delivery of the majority of therapeutics to the tumor [35–37].

**Fig. 1:**
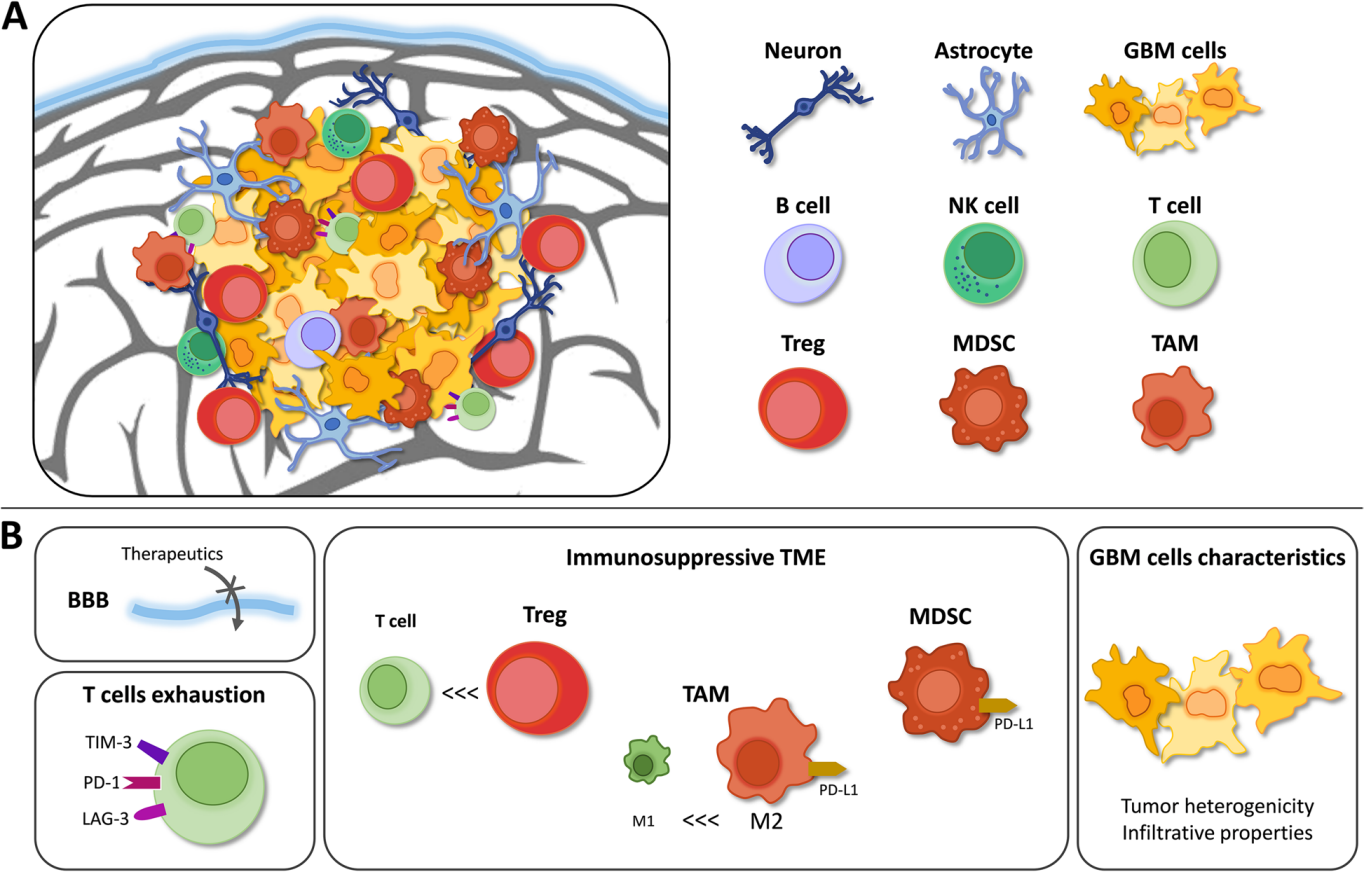
Schematic representation of GBM TME. **A** GBM TME is composed of various cell types. **B** Factors impeding translation of GBM immunotherapy treatments: (i) the BBB limiting drug delivery efficacy, (ii) the relatively low infiltration of T cells as well as their high exhaustion marker expression, (iii) the high infiltration of immunosuppressive cells (such as Tregs, TAMs and MDSCs) in the TME and (iv) the infiltrative and heterogeneous characteristics of GBM cells. (Abbreviations: GBM = Glioblastoma; LAG-3 = Lymphocyte activation gene 3 protein; MDSC = Myeloid-derived suppressive cell; NK = Natural killer; PD-1 = Programmed cell death-1; TAM = Tumor-associated microglia and macrophage; TIM-3 = T cell immunoglobulin and mucin domain containing-3)

Additionally, due to the presence of the BBB and its tight junctions, the brain has long been considered an immune-privileged site. The identification of an absence of classic lymphatics and tolerance of foreign tissue transplants in the brain also suggested that the brain was immunologically unique [38–40]. However, it is now known that the brain is not isolated immunologically [41]. It has been proven that functional lymphatic vessels are present in the CNS, that activated T cells can traffic to the CNS and that CNS antigens can reach the peripheral lymph nodes [38]. This emerging evidence suggests that immunotherapy can be applied to GBM and other brain cancers. However, several characteristics of GBM, e.g., its heterogeneity, BBB, low tumor mutation burden, low infiltration of T cells and microenvironment (which, for example, features a high infiltration of immunosuppressive cells) induce very complex immunosuppression, which is one major hindrance in finding new treatments and translating immunotherapies for GBM (Fig. 1).

GBM tumors develop in an immunosuppressive microenvironment that stimulates tumor cell growth and aggressiveness. The GBM TME is composed of various cell types: infiltrating tumor cells and cancer stem cells as well as noncancerous cells, such as myeloid cells (including resident microglia and bone marrow-derived macrophages), tissue-resident cells (such as neurons and astrocytes), and lymphocytes, and all these cells can interact together [42, 43].

GBM cells are known to secrete chemokines, growth factors and cytokines into the TME. Liberation of these molecules will attract and stimulate immunosuppressive cells [43–45]. In turn, immunosuppressive cells interact with GBM cells through different immunosuppressive receptors, resulting in tumor growth promotion and tumor cell resistance and evasion of immune surveillance [43, 44, 46, 47]. One of the most well-known and described immunosuppressive receptors involved in GBM immune escape is PD-1 [48–50]. The PD-1 receptor is mostly expressed on activated T cells, and binding of PD-1 with its ligand PD-L1 leads to inactivation of those T cells [51]. In GBM, PD-L1 has been shown to be overexpressed by GBM and myeloid cells, leading to effective binding of PD-L1 to PD-1 and therefore inhibition of the immune response [48, 52, 53].

The predominant non-neoplastic cells are tumor-associated microglia and macrophages (TAMs), which constitute approximately 30% of GBM TME [54, 55]. Microglia and macrophages are the main innate immune cells in the healthy CNS, where they play a major role in maintaining homeostasis and immune surveillance [56, 57]. Microglia are the resident macrophages of the CNS localized in the parenchyma. They arise from yolk-sac macrophage precursors at early embryonic stage [58–61]. Three non-parenchymal macrophage populations, or border-associated macrophages, are also found in the CNS under normal conditions: perivascular, meningeal and choroid macrophages [56, 57, 62]. In GBM, the partial disruption of the BBB leads to peripheral bone marrow-derived macrophages infiltration. This macrophage population account for approximately 85% of GBM TAMs [63].

Two TAM phenotypes are commonly described: (i) the classical inflammatory and anti-tumoral M1 phenotype and (ii) the anti-inflammatory and pro-tumoral M2 phenotype [64]. Recent studies also indicate that heterogeneous populations of TAMs expressing both M1- and M2-associated markers are found in human and murine GBM [65, 66]. A high infiltration of M2-like TAMs has been associated with poor prognosis in GBM [55, 67]. Moreover, a higher proportion of M2-like TAMs are found in high grade gliomas, as compared to low grade gliomas [68]. Indeed, GBM cells recruit microglia and macrophages in the TME by means of chemoattractants (e.g., C-C motif chemokine ligand 2 (CCL2), CSF-1) [69, 70] and induce a switch to pro-tumoral subtype [65, 71, 72]. In return, TAMs promote tumor cell proliferation and angiogenesis as well as inhibition of effector T cells proliferation and attraction of T regulatory cells (Tregs) and MDSCs through cytokines secretion [65, 72, 73].

MDSCs constitute a heterogeneous population of cells that play an important role in maintaining an immunosuppressive environment in GBM. They inhibit the immune response by interacting with different cells in the TME, promoting Treg function, limiting antigen presentation and inhibiting effector T cell activity, among other things [74–77]. In addition, as already mentioned, myeloid cells (including TAMs and MDSCs) have been shown to overexpress the negative checkpoint molecule PD-L1 in GBM patients, therefore promoting negative regulation of the immune response by inducing T cell dysfunction [78].

Compared to other tumors, the number of tumor-infiltrating lymphocytes (TILs) is relatively low in GBM [79]. Additionally, those that are present express high levels of exhaustion markers such as the inhibitory coreceptors T cell immunoglobulin and mucin domain containing-3 (TIM-3), lymphocyte activation gene 3 protein (LAG-3), and PD-1 [80–83]. Moreover, the fraction of Tregs among TILs is increased in GBM patients [84]. Tregs contribute to the TME immunosuppression by inhibiting effector T cells and antigen-presenting cells (APCs) [83]. Beside a low density of TILs, a low number of infiltrating Natural killer (NK) cells and B cells are found in GBM [85]. Besides, GBM cells have developed mechanisms to escape from NK immune surveillance through inhibitory bindings [86].

In addition to the complex interplay between all the cells present in the TME of GBM, resulting in multifactorial and complex immunosuppression, low tumor mutation burden is an additional likely reason why applying immunotherapy in GBM is difficult. Indeed, tumor-associated antigen production is quite low in GBM, which may lower the efficacy of immunotherapy [87].

Moreover, unique characteristics of GBM also impede the development of new treatments. Indeed, GBM is a very infiltrative tumor that exhibit inter- and intratumoral heterogeneity. Intratumoral heterogeneity refers to molecular diversity within a same tumor, leading to differences in growth rate, cellular morphology, histopathology and differentiation markers expression, among other things [88]. Therefore, different subpopulations of GBM cells coexist and cooperate to promote tumor growth and progression, contributing to divergence in response and resistance to SOC [89, 90]. Intertumoral heterogeneity refers to cellular and genetic differences between GBM tumors from different patients and leads therefore to different molecular subtypes, and heterogeneity in patients’ response to therapy [91].

## GBM immunotherapy: disappointing initial clinical results of monotherapies

Immune checkpoints are regulators of the immune system that control immune effector function by maintaining an equilibrium between inhibitory and costimulatory signals. Their role is to protect tissues from damage due to excessive immune response but also to prevent autoimmunity [14, 92]. However, cancer cells have been shown to take advantage of this system to escape immune surveillance by upregulating inhibitory immune checkpoint expression and activating these negative regulators on tumor-specific immune cells [14, 93, 94]. The main example of this mechanism in GBM is the appropriation of the PD-1/PD-L1 pathway [48–50].

The main immune checkpoints that have been successfully targeted in cancer treatments are PD-1, its ligand PD-L1, and cytotoxic T-lymphocyte antigen 4 (CTLA-4). Inhibition of these T cell negative regulators with mAbs increases the immune response in many cancers (e.g., melanoma and non-small-cell lung cancer) [25, 95–97]. In 2011, the FDA for the first time approved the use of the checkpoint inhibitor ipilimumab – a monoclonal antibody inhibiting CTLA-4 – as a frontline cancer treatment in advanced melanoma [22, 98]. Since 2014, the first mAb directed toward PD-1, nivolumab, has been FDA-approved for the treatment of metastatic melanoma [99]. Nivolumab was further approved for other cancers, such as non-small-lung cancer and squamous cancer of the head and neck [23, 24]. The combination of ipilimumab and nivolumab was also approved for advanced renal cell carcinoma, metastatic melanoma and metastatic colorectal cancer [100–102].

By generating and amplifying specific T cell responses against tumors, therapeutic cancer vaccines hold a major place among strategies to fight cancer. In the past decade, research on therapeutic cancer vaccine has extended, thanks to recent progress in delivery technologies and target selection methods as well as continuous advances in understanding tumor immune response mechanisms [103]. Today, there are 3 FDA-approved therapeutic cancer vaccines. The first immunotherapy ever approved by FDA was intravesical BCG (bacillus Calmette-Guérin) in 1990 [26]. This live attenuated vaccine is now part of the standard treatment of early-stage bladder cancer and reduces risks of cancer progression [104, 105]. In 2010, the FDA approved Sipuleucel-T (Provenge®) – an autologous dendritic cell (DC) vaccine that reduces the risk of death in prostate cancer patients [106]. Intralesional vaccination with an oncolytic herpes virus encoding GM-CSF, talimogene laherparepvec (T-VEC; Imlygic®), improved mOS in patients with advanced melanoma [107]. T-VEC was approved by the FDA in 2015 [108].

However, both ICBs and vaccines as monotherapies are still ineffective in multiple cancers, such as GBM. To date, there is no FDA-approved immunotherapy for GBM, even though some are being tested in clinical trials [109]. Moreover, some phase III clinical trials have failed, including trials of immunotherapy treatments such as ICBs or therapeutic vaccines (Table 1).

**Table 1:** Failures of phase III clinical trials of immunotherapy for GBM

Trial	Treatment	Outcome	Reference
CheckMate 143 phase III (NCT02017717)	**anti-PD-1** (nivolumab) vs anti-VEGF (bevacizumab)	**Primary endpoint not reached** ↔ No improvement of mOS with anti-PD-1 (9.8 months) vs anti-VEGF (10 months)	Reardon et al., (2020) [110]
CheckMate 498 phase III (NCT02617589)	**anti-PD-1** (nivolumab) + RT vs TMZ + RT	**Primary endpoint not reached** ↔ No improvement of mOS with anti-PD-1 + RT (13.4 months) vs control treatment (14.9 months)	BMS press release; ClinicalTrials.gov
CheckMate 548 phase III (NCT02667587)	**anti-PD-1** (nivolumab) + SOC vs placebo + SOC	**Primary endpoint not reached** Data not yet released	BMS press release
ACT IV phase III (NCT01480479)	**Peptide vaccine** targeting EGFRvIII (rindopepimut) + TMZ vs placebo + TMZ	**Primary endpoint not reached** ↔ No improvement of mOS with rindopepimut (20.1 months) vs control group (20.0 months)	Weller et al., (2017) [111]
NCT00045968 phase III	**Dendritic cell vaccine** (DCVax®-L) + SOC vs placebo + SOC	**Put on hold** For unidentified reasons	Liau et al., (2018) [112]; ClinicalTrials.gov

ICBs, and anti-PD-1 therapy more particularly, have been extensively studied in GBM treatment given their promising results in other solid tumors. Anti-PD-1 agents were the first type of ICB tested in a clinical trial for GBM treatment (CheckMate 143, NCT02017717) [110, 113]. Phase III of this study was conducted on 369 patients with recurrent GBM randomized to receive either nivolumab (anti-PD-1 antibody) or bevacizumab (an antibody targeting and inhibiting vascular endothelial growth factor (VEGF); an antiangiogenic treatment). The results of this study showed no improvement in the median overall survival (mOS) for patients treated with anti-PD-1 antibody compared to those in the other treatment arms [110]. Similarly, the phase III CheckMate 498 trial (NCT02617589) failed to meet its primary endpoint of improving mOS. The study was conducted on 560 newly diagnosed patients and compared nivolumab and TMZ, each of which was given with RT. More recently, Bristol-Myers Squibb (BMS) announced that the phase III trial Checkmate 548 (NCT02667587) did not meet its primary endpoint. The study evaluated the addition of nivolumab to the SOC and was conducted on 693 patients with newly diagnosed GBM.

Likewise, two phase III studies with therapeutic vaccines added to standard treatments failed to produce convincing results [111–113]. In the ACT IV phase III study (NCT01480479), the addition of rindopepimut – a vaccine targeting EGFRvIII – to standard chemotherapy did not improve survival of EGFRvIII-positive glioblastoma patients [111]. The NCT00045968 phase III trial evaluating the addition of an autologous tumor lysate-pulsed DC vaccine (DCVax®-L) to SOC for newly diagnosed GBM patients was put on hold by the FDA for unidentified reasons [112, 113].

The main commonality between the agents used in these disappointing phase III trials is that they stimulate only one arm of antitumor immunity: they reduce the immunosuppression exerted on T cells (ICBs), stimulate the immune response against a specific antigen or activate a specific dendritic cell (DC) response against the tumor. Even when combined with SOC, the immune response is not enhanced, mainly because of the immunosuppressive characteristics of systemic chemotherapy and RT (which induce lymphopenia and hypoxia, respectively), as well as corticotherapy [114, 115]. Indeed, the blood of patients treated with dexamethasone has a reduced number of immune cells [116].

Despite these disappointing clinical trial results, the randomized open-label pilot study from Cloughesy and colleagues raises hope for the use of immunotherapy in GBM [29]. In this study, an anti-PD-1 mAb (pembrolizumab) was given as a neoadjuvant drug to patients with recurrent GBM. Administration of pembrolizumab before resection significantly improved overall and progression-free survival with induction of TIL functional activation and production of an interferon (IFN)-γ response within the TME [29]. Besides, a phase II study on the therapeutic GBM vaccine ERC1671 (Gliovac™) showed promising preliminary results. ERC1671 is a vaccine composed of tumor cell lysate from allogeneic and autologous GBM patients mixed with primary irradiated/inactivated whole tumor cells [30]. Combination of ERC1671 with GM-CSF and cyclophosphamide plus bevacizumab resulted in a significant survival benefit with a mOS of 12 months, while placebo plus bevacizumab mOS was 7.5 months. Interim results suggested that the benefit was correlated with the CD4 helper T lymphocytes counts in the peripheral blood [30].

The use of immunotherapy for GBM is therefore not a dead end. According to us, combination strategies targeting different arms of the cancer immunity cycle have great potential to overcome GBM multifactorial immunosuppression and increase antitumor immune response. These strategies are being tested to a large extent in preclinical and clinical studies, giving encouraging results.

## Targeting different arms of the GBM immunity cycle to improve immunotherapy efficacy: preclinical aspects

**Fig. 2:**
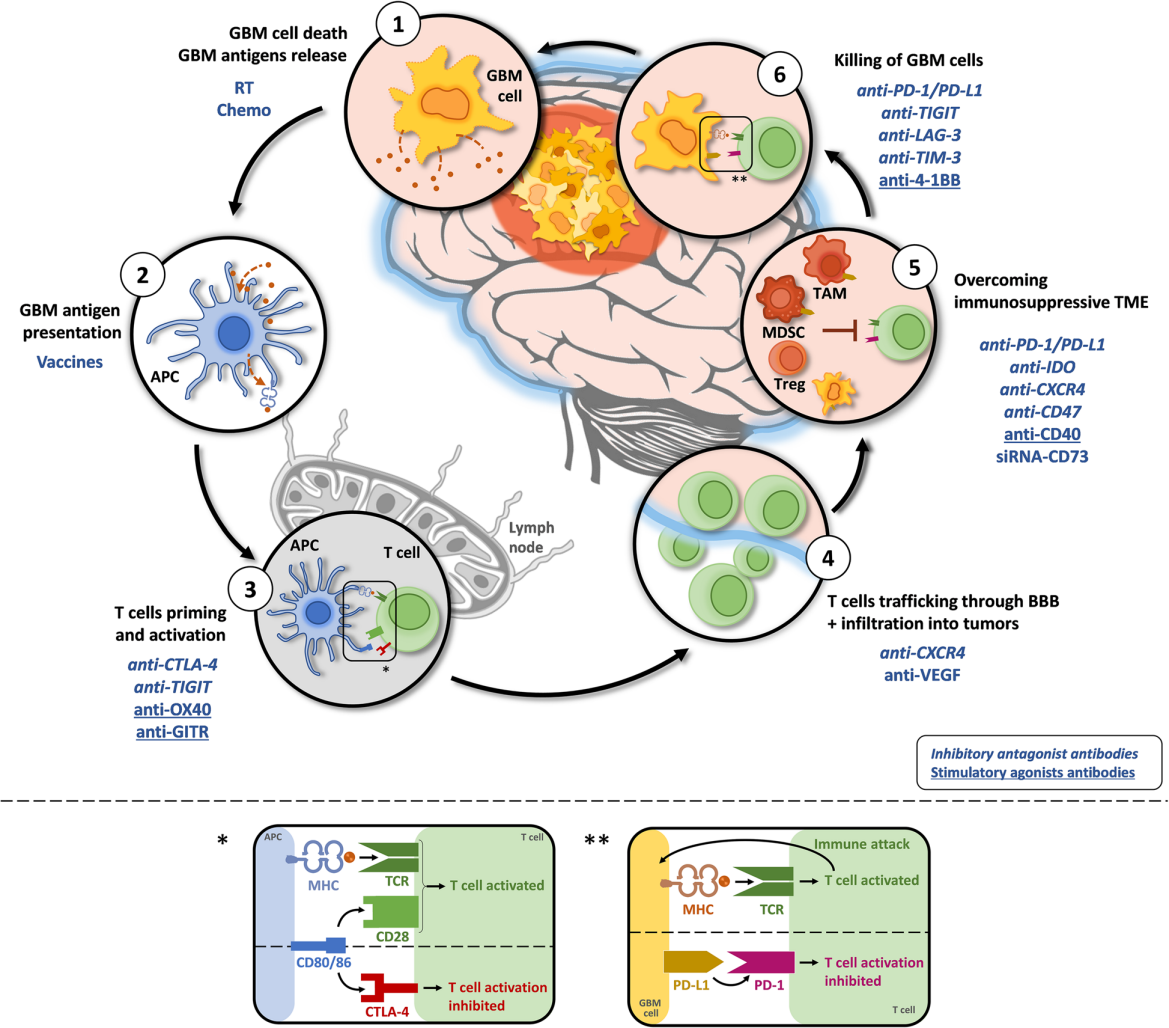
The GBM immunity cycle and associated treatments. The immune response in GBM can be divided into six steps, starting with antigen release from GBM cells and ending with the killing of GBM cells. Potential treatments impacting the immune response steps are written in blue. Step 1 – Antigens are released from dying GBM cells. Step 2 – Tumor antigens are captured by APCs, processed and displayed on MHC-I and -II molecules for presentation to T cells. Step 3 – Effector T cells are primed and activated in response to tumor antigen presentation. Step 4 – Activated T cells traffic through the BBB and infiltrate the tumor site. Step 5 – The immunosuppressive TME must be overcome to allow activated T cells to recognize and bind to GBM cells. Step 6 – Activated T cells kill GBM cells after binding to GBM tumor antigen on MHC-I through the T cell receptor (TCR). The boxes * and ** represent the CTLA-4 and PD-1/PD-L1 pathways. * T cells are activated after the binding of TCR with antigens displayed on MHC and the simultaneous CD28:CD80/86 costimulatory signal. CTLA-4 mediates T cell inhibition by competitively binding to CD80/86. ** T cells are activated after recognizing GBM cells, secreting inflammatory cytokines and inducing GBM cell death. PD-1:PD-L1 binding induces T cell inhibition by reducing T cell proliferation and cytokine production. (Abbreviations: APC = Antigen-presenting cell; Chemo = Chemotherapy; CTLA-4 = Cytotoxic T-lymphocyte antigen 4; CXCR4 = C-X-C chemokine receptor 4; GBM = Glioblastoma; GITR = Glucocorticoid-induced tumor necrosis factor-related protein; IDO = Indoleamine 2,3-dioxygenase; LAG-3 = Lymphocyte activation gene 3 protein; MDSC = Myeloid-derived suppressive cell; MHC = Major histocompatibility complex; PD-1 = Programmed cell death-1; PD-L1 = Programmed death ligand-1; RT = Radiotherapy; TAM = Tumor-associated microglia and macrophage; TCR = T cell receptor; TIGIT = T cell immunoreceptor with Ig and ITIM domains; TIM-3 = T cell immunoglobulin and mucin domain containing-3; VEGF = Vascular endothelial growth factor)

The immune cycle in GBM is a multistep process that can be targeted by treatments at different levels (Fig. 2). Antigens released from dying GBM cells are captured by APCs, processed and displayed on major histocompatibility complex (MHC)-I and -II molecules for presentation to T cells. Effector T cells are primed and activated in response to tumor antigen presentation. Activated T cells kill GBM cells after binding to GBM tumor antigen on MHC-I through the T cell receptor (TCR). However, the immunosuppressive microenvironment can hinder immune control in GBM.

ICBs act by restoring the activity of effector cells so that they can recognize and attack cancer cells again. However, if there is not enough T cell infiltration at the tumor site and/or too many immunosuppressive cells in the TME (e.g., Tregs and TAMs), ICBs may not be enough to promote a strong antitumor response inducing the death of cancer cells. Targeting different arms of the cancer immunity cycle is of great interest, particularly in the context of GBM, in which it could offer better chances to overcome multifactorial immunosuppression [117, 118]. Indeed, it is possible to stimulate the immune system at different times and locations during immune response generation. One possibility to enhance the immune response against GBM (or other cancers) is by stimulating antigen release from dying tumor cells and their presentation to APCs. Immunogenic cell death (ICD) inducers, such as RT and some chemotherapeutics, induce dying cancer cells to release danger signals that stimulate the recruitment of APCs and antigen presentation [117]. Antigen presentation may also be stimulated by using vaccines (e.g., whole-cell tumor vaccines or peptide vaccines) that enhance the recognition of antigens and increase the production of antigen-specific T cells [118]. Another way to improve the immune response against GBM is to decrease the immunosuppression occurring at different stages of the generation of effector T cells. The use of ICBs is helpful to block the inhibition of effector T cells and therefore restore their activity (either early in the course of their activation or in the TME) [119]. Costimulatory agonist mAbs might also be of great interest to potentiate effector T cell function [120] (Fig. 2).

In this chapter, we describe some of the combination strategies that are used in preclinical studies. Most of the described results rely on GL261(−Luc) models, as they are the most common models used nowadays for studying the immunotherapy of GBM. We focus first on combination of chemotherapy with immunotherapy and then on combination of different immunotherapies. ICBs are the most studied immunotherapeutic approach; for this reason, the majority of the combinations presented hereafter includes inhibitory checkpoint molecules. However, we also describe other strategies, including vaccines and agonist costimulatory checkpoints.

### Combination of immunotherapy with chemotherapy

The combination of ICBs with chemotherapy, particularly TMZ, is one of the most studied combination therapies for GBM. The combination of chemotherapy and ICBs offers the advantage of enhancing the recognition and elimination of tumor cells (e.g., by increasing tumor antigenicity, inducing ICD and reducing the immunosuppression exerted on effector cells in the TME) [121, 122]. Moreover, as TMZ is part of the SOC, the combination could have potential for inducing a first-line antitumor immune response [13]. However, a standard dose of TMZ causes severe lymphopenia and T cell exhaustion, and anti-PD-1 immunological benefits are almost totally absent. Although the combination of systemic administration of a standard dose of TMZ and anti-PD-1 mAbs has been shown to increase the survival rate in murine GBM, no benefit of immune modulation has been observed, and tumors recur [123–125] (Table 2).

**Table 2:** Combinations of immunotherapy and chemotherapy in preclinical GBM models

Combination treatment	Protocol	Cell line and model	Outcome	Reference
anti-PD-1 TMZ	• Tumor implantation: 2 × 10^5^ cells • TMZ: 30 mg/kg, 5 consecutive days starting at d8, IP • anti-PD-1: 10 mg/kg 2x on d13 and d15, IV	GL261-Luc orthotopic syngeneic	• Combined therapy showed better antitumor efficacy than monotherapies with 100% tumor regression • TMZ abrogated the favorable immunological effects of anti-PD-1 (increased TIL numbers, decreased Treg and exhausted T cell frequencies, increased immunological memory)	Park J., et al. (2018) [123]
anti-PD-1 TMZ (standard or metronomic dose)	• Tumor implantation: / • TMZ: - Standard dose (SD): 50 mg/kg for 5 consecutive days, IP - Metronomic dose (MD): 25 mg/kg for 10 consecutive days, IP • anti-PD-1: 10 mg/kg 4x every 5 days, IP	GL261 orthotopic syngeneic	• SD TMZ increased exhaustion markers on T cells, while MD TMZ did not lead to T cell exhaustion • anti-PD-1 reversed the exhaustion induced by SD TMZ in peripheral T cells but not in TILs • The survival benefit of anti-PD-1 therapy was abrogated by SD TMZ but not by MD TMZ	Karachi A., et al. (2019) [124]
anti-PD-1 TMZ (low dose)	• Tumor implantation: 5 × 10^4^ cells, right cerebral cortex • TMZ: 50 μg/kg, 5 consecutive days, IP • anti-PD-1: 200 μg 3x, IP	GL261 orthotopic syngeneic	• Combined therapy synergistically inhibited GBM tumor growth with a higher median survival time, a reduced tumor volume and 40% long-term survivors • Combined therapy increased CD4 and CD8 T cell infiltration in tumor lesions	Dai B., et al. (2018) [126]
anti-PD-1 TMZ or carmustine (BCNU) (systemic or local administration)	• Tumor implantation: 1.3 × 10^5^ cells, left striatum • Systemic chemotherapy (SC): - TMZ: 66 mg/kg, daily from d10 to d14, IP - BCNU: 5, 15 and 30 mg/kg, 3x/week for 2 weeks starting at d14, IP • Local chemotherapy (LC): - TMZ: implanted at d10 - BCNU: implanted at d14 Polymer impregnated with chemotherapy (wafer), allowing constant release in the TME for at least 2 weeks, placed directly on top of the tumor • anti-PD-1: 200 μg 3x, on d0, d12, and d14, IP	GL261-Luc orthotopic syngeneic	• Combination of LC and anti-PD-1 induced a robust immune response and survival benefit, with higher numbers of TILs and IFN-γ-secreting CD8 T cells in the brain, a higher Teff/Treg ratio and a higher tumor-infiltrating DC % • LC preserved the memory response upon rechallenge; SC abrogated it • SC abrogated the immunological benefits of anti-PD-1, did not provide survival benefit and resulted in severe lymphodepletion and severe depletion of TILs • SC alone or in combination with anti-PD-1 delayed tumor progression, but tumors recurred	Mathios D., et al. (2016) [125]
TMZ (systemic) ICD-based DC vaccine	• Tumor implantation: 5 × 10^5^ cells • ICD-based DC vaccine: 1 × 10^6^ DCs, IP - On d2, d9 and d15 – vaccine alone - On d21, d28 and d35 – combination Production: cancer cells were incubated with hypericin followed by light irradiation, and then, Hyp-PDT-treated cells were mixed with DCs • TMZ: 40 mg/kg, 6x on d5, d7, d9, d12, d14, and d16	GL261 orthotopic syngeneic	• Combined therapy provided a strong survival benefit with improved median survival and 50% long-term survivors • The ICD-based vaccine partially overcame the immune-ablating effects of chemotherapy. TMZ decreased the levels of brain-infiltrating CD8 T cells, but the combination decreased the levels of Tregs in the brain	Garg AD., et al. (2016) [127]
TMZ (systemic or local) GL-GM (whole tumor cell vaccine)	• Tumor implantation: 5 × 10^3^ cells, right frontal lobe • GL-GM: 2 × 10^6^ irradiated (40 Gy) GL261-GMCSF cells, on d5, d19, and d33, IP • TMZ: - Systemic (SC): 50 mg/kg, at d7, d8 and d9, IP - Local (LC): 4.2 mg/kg/day, from d7 to d9, intratumoral	GL261 orthotopic syngeneic	• Local administration of TMZ induced a higher survival rate than systemic administration, and the effect was T cell-dependent • SC but not LC TMZ depleted blood leukocytes • Combination of TMZ IC and GL-GM increased survival and induced immune benefits with increased CD4 and CD8 TILs • Immune memory was established in long-term survivors (SC TMZ + GL-GM)	Fritzell S., et al. (2013) [128]
TMZ (local) Whole cell vaccine	• Tumor implantation: 5 × 10^3^ cells, right frontal lobe • Whole cell immunization: 2 × 10^6^ irradiated (40 Gy) cells (GL261 or KR158) on d5, d19, and d33, SC • TMZ: 180 μg administered over 3 days, starting on d7, convection-enhanced delivery (CED), intratumoral	GL261 or KR158-Luc orthotopic syngeneic	• CED-TMZ and the whole cell vaccine synergized in the GL261 model resulting in 93% long-term survivors • The whole cell vaccine cured some mice of the KR158 model, and CED-TMZ prolonged median survival; however, there was no synergy between chemotherapy and immunotherapy • CED-TMZ plus the vaccine significantly decreased tumor volume and increased the intratumoral influx of T cells in both models	Enriquez Pérez JE., et al. (2020) [129]
TMZ anti-CD47 anti-PD-1	• Tumor implantation: 1 × 10^5^ cells, caudate putamen • TMZ: - Concurrent: 80 mg/kg, at d11, d13 and d15, IP - Sequential: metronomic dose (20 mg/kg) at d7–9 + 80 mg/kg at d11, d13 and d15, IP • anti-CD47 (MIAP-140): 100 μg, at d11, d13 and d15, IP • anti-PD-1: 100 μg, on d16, d18 and d20, IP	GL261 or CT2-A orthotopic syngeneic	• Sequential TMZ treatment combined with anti-CD47 improved tumor growth inhibition and mice survival; monotherapies and concurrent treatment did not • Combination of sequential TMZ and anti-CD47 activated immune response in vivo, with significant increase of CD4 and CD8 T cell, IFN-γ-secreting cell and activated TAM numbers • Triple combination of TMZ, anti-CD47 and anti-PD-1 further improved the survival	von Roemeling CA., et al. (2020) [130]

One way to enhance ICBs efficacy while using chemotherapy is to modify the dose or the administration route (Table 2). It has in fact been proven that metronomic doses of TMZ do not induce exhaustion of T cells, while standard doses lead to upregulation of exhaustion markers [124]. Moreover, the anti-PD-1 survival benefit was maintained upon the addition of metronomic doses of TMZ, while it was abrogated upon the addition of standard TMZ doses [124]. Comparatively, low doses of TMZ combined with anti-PD-1 therapy also led to survival benefits with an increased number of TILs [126].

Modifying the administration route has also proven to improve the efficacy of combined chemotherapy and immunotherapy, as shown by Mathios and colleagues [125]. In their study, they demonstrated that local administration of chemotherapy, using a wafer impregnated with either TMZ or carmustine (bis-chloroethylnitrosurea (BCNU)), enhanced anti-PD-1 antitumor effects, while systemic chemotherapy abrogated them [125]. One potential explanation for the synergistic effect observed with local chemotherapy is the increased antigen presentation during chemotherapy-induced tumor cell death. This explanation was supported by an immune profile analysis, which showed an increased percentage of DCs in groups treated with local chemotherapy [125].

Similar trends have been observed when TMZ is combined with vaccines in preclinical studies using GBM murine models (Table 2). The preclinical study of Garg and colleagues in 2016 also proved the immune ablative effects of systemic TMZ [127]. The researchers showed a survival benefit after treatment with TMZ combined with an ICD-based DC vaccine. However, the addition of TMZ decreased the levels of infiltrating CD8 T cells [127]. Additionally, a comparison between systemic and intratumoral administration of TMZ in combination with a whole-cell tumor vaccine showed better survival and immune benefits with local administration of chemotherapy [128]. Moreover, while lymphodepletion was observed following systemic administration, there was none after local administration [128]. Finally, the importance of combining immunotherapy with local chemotherapy was again proven in a study using both the GL261 and KR158 glioma models. Therapy with a whole-cell tumor vaccine combined with intratumoral convection-enhanced delivery (CED) of TMZ not only increased the number of long-term survivors and reduced tumor volume but also increased TILs in both models [129].

More recently, studies have also been focusing on immunotherapy targeting innate immune checkpoints given the key role of the innate immune system in the early detection of cancer as well as in the initiation and maintenance of an immune response [131]. CD47 is expressed on the cell surface of solid tumor cells and its ligand, signal regulatory protein alpha (SIRPα), is expressed on macrophages and DCs [132]. CD47 acts like an antiphagocytic signal for phagocytic cells; blockade of CD47 with mAbs induce therefore macrophage phagocytosis of cancer cells [133]. The effects of the innate ICB anti-CD47 in combination with TMZ was evaluated on GL261 and CT-2A mouse models [130]. Combination of TMZ and anti-CD47 mAbs inhibited tumor growth and significantly improved the survival by activating both innate and adaptive immune responses. Indeed, activation of the cyclic GMP-AMP synthase-stimulator of interferon genes (cGAS-STING) pathway and increased numbers of activated macrophages as well as higher numbers of T cells and IFN-γ-secreting CD8 T cells were observed in mice with GBM [130]. The combination was further improved by adjuvant PD-1 blockade, probably because it helped overcoming adaptive immune resistance [130]. It is worth noting that schedule of TMZ administration was once more of major importance in this study. While concurrent TMZ/anti-CD47 treatment did not induce survival and immune benefits, sequential treatment with metronomic doses of TMZ administered before the concomitant TMZ/anti-CD47 did [130].

### Combination of multiple immunotherapies

Combination of different ICBs offers the opportunity to enhance their efficacy and is often studied in many cancers [93]. Indeed, not all immune checkpoints act on effector T cells at the same location or time in the course of their activation (Fig. 2) [134]. Therefore, the use of different ICBs may result in synergistic effects [134, 135]. Moreover, while ICB as a monotherapy induces compensatory upregulation of other immune checkpoints, numerous preclinical studies of different cancers have demonstrated better outcomes and tumor growth decreases with the inhibition of two or more checkpoint receptors [82, 134–136]. In preclinical studies of murine GBM, combinations of multiple ICBs or combinations of ICBs with other immunotherapies have been shown to increase the immune response and survival rate (Table 3).

**Table 3:** Combinations of immunotherapies including ICB in preclinical GBM models

Combination treatment	Protocol	Cell line and model	Outcome	Reference
anti-PD-1 anti-LAG-3	• Tumor implantation: 1.3 × 10^5^ cells, striatum • anti-PD-1: 200 μg, IP - on d7, d10, d12, and d14 (early schedule) - on d10, d12, and d14 (late schedule) • anti-LAG-3: 200 μg, IP - on d7 and d10 (early schedule) - on d10 and d12 (late schedule)	GL261-Luc orthotopic syngeneic	• Early treatment with anti-LAG-3 and/or anti-PD-1 significantly improved survival • Late treatment with anti-LAG-3 did not significantly improve survival, but the combination did • The global immunological profiles were not different between the different treatment arms • Immune memory was established in long-term survivors	Harris-Bookman S., et al. (2018) [137]
anti-PD-1 anti-TIGIT	• Tumor implantation: 1.3 × 10^5^ cells, striatum • anti-PD-1: 200 μg, on d10, d12, and d14, IP • anti-TIGIT: 200 μg, IP, every other day for a total of 5 doses starting on d8, d10, d12 or d14 (4 ≠ schedules: A, B, C, D)	GL261-Luc orthotopic syngeneic	• Combination therapy improved long-term survival following each schedule • Combination therapy increased immune cell tumor infiltration and cytokine production • Tumor-infiltrating DCs were reduced following anti-TIGIT and anti-PD-1 combination treatment • Immune memory was established in long-term survivors	Hung AL., et al. (2018) [138]
anti-CTLA-4 anti-PD-L1 1-MT	• Tumor implantation: 4 × 10^5^ cells • anti-CTLA-4: 100 μg loading dose followed by 3 × 50 μg maintenance doses every 3 days, IP • anti-PD-L1: 500 μg loading dose followed by 3 × 200 μg maintenance doses every 3 days, IP • 1-MT: in the drinking water over 30 days, starting on d7 for early blockade or on d14 for late blockade	GL261 orthotopic syngeneic	• Early blockade with the triple combination cured 100% of mice, reduced Treg infiltration and increased IFN-γ-secreting CD8 T cell infiltration • Late blockade prolonged survival (78% long-term survival rate) and reduced Treg infiltration but also reduced brain-infiltrating T cells	Wainwright DA., et al. (2014) [139]
anti-GITR agonist SRS	• Tumor implantation: 1.3 × 10^5^ cells, striatum • SRS: 10 Gy radiation (1.9 Gy/min), d10, focal • anti-GITR: 10 mg/kg, 3x, on d10, d13, and d16, IP	GL261-Luc orthotopic syngeneic	• Combination therapy improved survival • The combination increased the CD8 effector T cell/Treg ratio • Immune memory was established in long-term survivors	Patel MA., et al. (2016) [140]
anti-PD-1 anti-OX40 agonist GVAX (whole tumor cell vaccine)	• Tumor implantation: 7.5 × 10^4^ cells, right striatum • anti-PD-1: 200 μg, on d3, d6, and d9, IP • anti-OX40: 250 μg, on d3, d6, and d9, IP • GVAX: 1 × 10^6^ irradiated (35 Gy) GL261-GMCSF cells, on d3, d6, and d9, SC	GL261 orthotopic syngeneic	• The anti-PD-1 + anti-OX40 dual combination improved survival and the CD8/Treg ratio • The anti-PD-1 + GVAX dual combination improved survival and the CD8/Treg ratio and increased brain-infiltrating CD8 T cells • The triple combination led to 100% long-term survival with an increase in IFN-γ- and IL-2-secreting splenocytes and the CD4/CD8 ratio • Immune memory is established in long-term survivors	Jahan N., et al. (2019) [141]
anti-PD-L1 Neoantigen peptide vaccine	• Tumor implantation: 5 × 10^4^ cells • anti-PD-L1: on d7, d9, and d11, IP • Vaccine: 50 μg of each peptide + 100 μg polyIC adjuvant, on d3, d6, and d9, SC	CT2A orthotopic syngeneic	The combination therapy significantly improved mouse survival (60% long-term survivors)	Liu CJ., et al. (2020) [142]
anti-PD-1 anti-TIM3 SRS	• Tumor implantation: 1.3 × 10^5^ cells, left striatum • SRS: 10 Gy radiation (1.9 Gy/min), d10, focal, using the Small Animal Radiation Research Platform (SARRP) • anti-PD-1: 200 μg, on d10, d12, and d14 • anti-TIM-3: 250 μg, on d7, d11, and d-15	GL261-Luc orthotopic syngeneic	• The anti-PD-1 and dual therapies improved survival and led to long-term survival • The triple combination led to 100% remission • The triple combination improved the immune profile of the TME and the cytokine profile of both CD4 and CD8 T cells (increased the CD8/Treg ratio, decreased the frequency of FoxP3+ Tregs, and increased the production of the inflammatory cytokines IFN-γ, TNF-α, and IL-17a) • Immune memory was established in long-term survivors	Kim JE., et al. (2017) [143]
anti-CTLA-4 anti-4-1BB agonist SRS	• Tumor implantation: 1.3 × 10^5^ cells, left striatum • SRS: 10 Gy radiation (1.9 Gy/min), d10, focal, using the SARRP • anti-4-1BB: 200 μg 3x, on d11, d14, and d17, IP • anti-CTLA-4: 800 μg 3x, on d11, d17, and d23, IP	GL261-Luc orthotopic syngeneic	• The SRS + anti-CTLA-4 dual therapy prolonged survival, but only the triple combination led to long-term survival • The triple therapy and double immunotherapy led to higher TILs (CD4 and CD8 T cells) • Immune memory was established in long-term survivors and was glioma-specific	Belcaid Z., et al. (2014) [144]
anti-PD-1 anti-CXCR4	• Tumor implantation: 1.3 × 10^5^ cells, left striatum • anti-PD-1: 200 μg, on d10, d12, and d14, IP • anti-CXCR4: 200 μg, on d10, d12, and d14, IP	GL261-Luc orthotopic syngeneic	• The combination improved survival • Combination therapy decreased populations of suppressive myeloid cells in the brain • Combination therapy decreased the CD4/CD8 and Treg/CD8 ratios in the brain to a higher extent than did the monotherapies • The combination and monotherapies increased the levels of circulating inflammatory antitumor cytokines • Immune memory was established in long-term survivors	Wu A., et al. (2019) [145]
anti-PD-1 Antiangiogenic therapy (anti-VEGF + anti-Ang-2)	• Tumor implantation: 1 × 10^5^ cells, striatum • anti-PD-1: 10 mg/kg, 2x/week starting on d10 for a total of 8 doses, IP • Antiangiogenic therapy: 25 mg/kg (anti-VEGF) and 5.6 mg/kg (anti-Ang-2), 2x/week starting on d5 until symptom apparition, SC	GL261 orthotopic syngeneic	• The triple therapy significantly improved survival compared to antiangiogenic therapy alone • The triple therapy increased CD8 TIL numbers and decreased MDSCs and Tregs in the brain The antiangiogenic therapy efficacy was improved by the addition of anti-PD-1 therapy	Di Tacchio M., et al. (2019) [146]
anti-PD-1 anti-CTLA-4 G47Δ-mIL12 (= Oncolytic herpes simplex virus expressing IL-12)	• Tumor implantation: 2 × 10^4^ cells (005 GSCs) or 1 × 104 cells (CT-2A), striatum • anti-PD-1: 10 mg/kg, on d8, 11 and d14 (005 GSCs) or on d10, d13 and d16 (CT-2A), IP • anti-CTLA4: 5 mg/kg, on d8, d11 and d14 (005 GSCs) or on d10, d13 and d16 (CT-2A), IP • G47Δ-mIL12: 5 × 10^5^ PFU, on d8 (005 GSCs) or on d10 (CT-2A), intratumoral	mouse 005 GSCs or CT-2A orthotopic syngeneic	• Individual ICB only minimally extended survival in 005 GBM; combination of G47Δ-mIL12 with individual ICB modestly improved efficacy • G47Δ-mIL12 decreased the % of 005 cells and Tregs and induced M1-like polarization in TAMs; these effects are further increased with the triple combination • Dual ICBs significantly increased CD8 T cells in the brain, triple combination increased CD8 T cells and decreased Tregs • Triple combination resulted in 89% or 50% long term survival (005 GSCs or CT-2A, respectively) Immune memory was established in long-term survivors	Saha D., et al. (2017) [147]
anti-PD-1 anti-CTLA-4 anti-IL-6 anti-CD40 agonist	• Tumor implantation: 3 × 10^5^ genetically engineered mouse tumor cells or 2 × 10^5^ GL261 cells • anti-PD-1: 200 μg on d9, d12, d15 and d18, IP • anti-CTLA-4: 200 μg on d9, d12, d15 and d18, IP • anti-IL-6: 200 μg on d9, d12, d15 and d18, IP • anti-CD40: 100 μg on d12, IP	RCAS-genetically engineered model or GL261 orthotopic syngeneic	• Dual targeting of IL-6 and CD40 sensitized GBM to ICBs; survival was significantly improved following triple combination • Dual targeting of IL-6 and CD40 reduced tumor growth, triple combination blocked it • All treatments induced a decrease in immunosuppressive TAM activity • All treatments, except anti-CD40 alone, decreased the expression of immunosuppressive cytokines (IL-10, TGFβ) • Only triple combination induced an increase in TILs and in IFN-γ-secreting CD8 T cells	Yang F. et al. (2021) [148]

The expression of LAG-3 by CD4 and CD8 T cells was correlated with a significant decrease in their IFN-γ production, corroborating other study results showing that LAG-3 is a marker of T cell exhaustion [137]. LAG-3 is a receptor upregulated on activated NK and T cells, and binding to MHC-II – its main ligand – induces negative regulation of T cells by decreasing proliferation and cytokine production [149–151]. The combination of anti-LAG-3 with anti-PD-1 significantly improved long-term survival. The combination was more effective when anti-LAG-3 was given at an early point. However, no difference in the immunological profile was observed when comparing the combination therapy and the other treatment arms [137].

The expression of the checkpoint molecule T cell immunoreceptor with Ig and ITIM domains (TIGIT) was found to be upregulated on CD8 T cells and Tregs in the brains of mice bearing GBM tumors compared to the expression seen in lymph nodes and spleens [138]. TIGIT is a negative checkpoint receptor that is mostly upregulated by NK and T cells, and its ligands are mainly expressed by tumor cells and APCs. TIGIT pathways induce, among other things, negative regulation of T cell-mediated tumor recognition and promotion of NK cell–dependent tumor immunity in different mouse models [152, 153]. Treatment of mice with a combination of anti-TIGIT and anti-PD-1 mAbs significantly improved survival compared to their treatment with monotherapies, with an increase in effector T cell function and downregulation of immunosuppressive cells [138].

The combination of dual ICB therapy with 1-methyltryptophan (1-MT) – an inhibitor of the tryptophan catabolic enzyme indoleamine 2,3-dioxygenase (IDO) – significantly improved the survival of mice bearing intracranial GBM tumors [139]. The inhibition of PD-L1, CTLA-4 and IDO synergistically decreased Treg infiltration. Early blockade of these checkpoints induced an increase in effector CD8 T cell infiltration in the brain and led to 100% long-term survival. Late blockade, however, induced a decrease in TILs and led to cure of 78% of the mice [139].

In addition to inhibitory checkpoint molecules, agonists of costimulatory checkpoint pathways are also promising in the research of new immunotherapy treatments [154]. Treatment of mice with stereotactic radiosurgery (SRS) and a glucocorticoid-induced tumor necrosis factor-related protein (GITR) agonist – which induces stimulation of effector T cells and inhibition of Tregs [155, 156] – induced long-term survival, with an increase in the CD4 T cell/Treg ratio as well as elevated cytokine production by CD4 and CD8 TILs [140]. Combination of costimulatory agonist mAbs with ICB has also shown efficacy in GBM murine models. While using ICB reduces the immunosuppression of effector cells, targeting costimulatory receptors increases effector cell activity, and the combination of both approaches could reinforce the immune response against cancers [135].

The efficacy of combining an anti-PD-1 antibody with an agonist anti-OX40 antibody has been demonstrated, with an increase in the long-term survival rate and the CD8 T cell/Treg ratio in the brain [141]. A triple combination of anti-PD-1 immunotherapy, an anti-OX40 agonist and GVAX – a whole tumor cell vaccine – induced cure of 100% of mice [141]. The synergistic effect was due to complementary actions of the three treatments. Indeed, while GVAX increased the number of activated tumor-specific T cells and infiltrating CD8 T cells, PD-1 blockade further stimulated them, and OX40 induced a vigorous type 1 helper T (Th1) cell response and decreased Treg infiltration [141].

Likewise, neoantigen vaccination has shown synergistic effects when combined with ICB in a CT-2A orthotopic model [142]. Three newly identified neoantigens were used in a polyvalent peptide vaccine and tested in combination with anti-PD-L1 treatment. Following combination therapy, survival was significantly improved compared to that achieved by monotherapies [142].

Other triple therapies targeting different steps of the cancer-immunity cycle have also shown more encouraging results than dual therapies, with a significant improvement in survival and immune profile. The combination of anti-PD-1 mAbs, anti-TIM-3 mAbs and SRS cured 100% of mice. This combination improved the TME immune profile, with an increase in both the CD8 T cell/Treg ratio and the number of IFN-γ-producing CD4 and CD8 TILs [143]. The combination of anti-CTLA-4 mAbs with SRS and an agonist of 4-1BB – a T cell costimulatory checkpoint inducing activation, proliferation and cytokine production [155, 157] – significantly prolonged the survival of mice and increased the number of long-term survivors [144]. The authors demonstrated an increase in TILs and a glioma-specific memory response. The antitumor activity of this triple combination was shown to be CD4 T cell-dependent [144].

Combinations of ICBs with immunotherapies or treatments decreasing GBM TME immunosuppression has also shown synergistic effects. An anti-PD-1 and anti-C-X-C chemokine receptor 4 (CXCR4) combination improved the survival of GBM-bearing mice [145]. CXCR4 overexpression in GBM contributes to treatment resistance through recruitment of immunosuppressive myeloid cells and promotion of tumor aggressiveness [145, 158, 159]. It was demonstrated that targeting myeloid cells with anti-CXCR4 enables anti-PD-1 therapy to induce an antitumor immune response [145].

Modulation of angiogenesis is another way to target the TME. While antiangiogenic therapy and anti-PD-L1 mAbs as monotherapies have both failed in improving the survival of GBM patients, a preclinical study showed that combining both approaches could improve the efficacy of GBM immunotherapy. Anti-VEGF/Ang-2 therapy followed by anti-PD-L1 treatment decreased MDSCs and Tregs in the brain, increased effector CD8 T cell infiltration and improved survival [146].

Targeting the adenosinergic pathway, that has recently been discovered as a major actor in GBM TME immunosuppression, also showed promising preclinical results [160, 161]. Blockade of CD73 – an ectonucleotidase converting ATP to adenosine and involved in chemoresistance and tumor invasion and proliferation [162–164] – decreased GBM growth and modulate GBM TME by reducing TAMs and Tregs infiltration [165]. Moreover, silencing CD73 improved survival of mice treated with ICBs [162].

Strategies targeting TAMs in GMB TME in combination with ICBs are also being explored. It was demonstrated that TAMs polarization promoted eradication of GBM tumors following combination of ICBs and immunovirotherapy [147]. G47Δ-mIL12 – an oncolytic herpes simplex virus expressing murine IL-12 – induced M1 polarization of TAMs, plausibly because of IFN-γ expression induced by IL-12. The effect of G47Δ-mIL12 was further increased with the addition of anti-CTLA-4 and anti-PD-1 due to an influx of TAMs. The triple combination also induced an increase in effector CD8 T cells. Altogether, triple combination synergistic effects led to the cure of 89% of 005-GSCs-bearing mice and 50% of CT-2A-bearing mice [147]. What is worthy of note is that combining the oncolytic virus with only one ICB was not sufficient to induce long-lasting effects and overcome GBM immunosuppression [147].

Likewise, it was demonstrated that inhibition of IL-6 reversed TAMs-mediated immunosuppression. However, IL-6 inhibition also induced a reduction in CD40 expression, leading to treatment resistance [148]. CD40 is expressed on APCs (DCs and macrophages) and ligation with CD40L on T cells activates both T cells and APCs, by increasing the latter antigen-presenting and co-stimulatory functions [166, 167]. Anti-CD40 agonist mAbs were able to reprogram the TME and synergized with ICBs in other cancers [168–170]. But in GBM preclinical models, agonist CD40 monotherapy did not demonstrate therapeutic improvement [148, 171]. However, combination of IL-6 inhibition and CD40 activation reversed TAMs-mediated GBM immunosuppression and sensitized GBM to anti-PD-1 and anti-CTLA-4 [148].

## Limitations of current preclinical models and future outlook to improve combination strategies for the immunotherapy of GBM

Many immunotherapy strategies that have shown successful results in preclinical studies have failed to produce convincing results in clinical trials, revealing limitations and inadequacies of current GBM preclinical models [110, 111, 172]. Translational impact may be improved by developing new relevant preclinical models. In the literature, murine GL261 GBM cell lines are the most commonly used. However, they are highly immunogenic unlike human GBM [172, 173]. Additionally, luciferase-expressing GL261 cells display even more immunogenic features than GL261, with a prolonged median survival time and an elevated inflammatory cytokines production [174]. Transitioning to other models that are less immunogenic and closer to human GBM is highly encouraged [175]. To that purpose, the recently developed SB28 and 005 GSC models are interesting alternatives and are among the best syngeneic models to represent human GBM TME [147, 171, 176–178]. Indeed, they are poorly immunogenic, with absence of MHC molecules expression as well as low immune cell infiltration and activation [177–179]. These models are moreover resistant to ICBs [147, 178].

An alternative to syngeneic immunocompetent mouse models is the use of genetically engineered mouse models (GEMs). GEMs of GBM reproduce more closely the histology and biology as well as the development of human GBM [172, 180]. Another advantage of these models is that they usually do not require intracranial injections as they are generated through genetic modifications [180]. However, GEMs still do not reflect GBM heterogeneity. Moreover, setting up GEMs requires a lot of expertise and can be expensive [172].

Using patient-derived xenograft models is the best approach to gather human GBM histology and heterogeneity as well as intratumoral heterogeneity [172]. However, these kinds of models generally require immunodeficient mice, which are not suitable for immunotherapy studies [181]. Yet, an immunocompetent mouse model of human GBM was recently developed. By transiently blocking T cell costimulation, the researchers managed to grow human GBM xenograft while keeping intact the mouse immune system [182]. This could be a step forward in the preclinical research for immunotherapy against GBM.

However, the perfect model imitating exactly all characteristics of human GBM (e.g., intratumoral heterogeneity, invasive properties, low immunogenicity, resistance to radio- and chemotherapy) doesn’t exist [172, 175]. It is therefore crucial to design the experiment and select the models according to their unique characteristics. In addition, studies on different models will help to develop a more general therapy against GBM by (i) covering multiple aspects of GBM immunology and (ii) addressing GBM heterogeneity (intratumoral and interpatient) [175].

Nevertheless, to improve immunotherapy for GBM, novel strategies need to be studied. Looking at the development of novel therapeutic targets (e.g., STING, Toll-like receptor (TLR) agonists or old combination therapies applied to new targets) is important to assess the utility of targeting underexplored pathways useful for improving combination immunotherapy [183–187]. In pursuit of this goal, targeting immunosuppressive factors and/or cells within GBM TME is highly valuable [39]. To do so, targeting angiogenesis signaling factors (e.g., VEGF, Ang-2) [146] and adenosinergic pathways components (e.g., adenosine receptors, CD73, CD39) [160, 165, 173, 188] has shown great potential in preclinical studies. Moreover, targeting TAMs are particularly interesting since these cells are the major non-neoplastic cellular components of GBM TME. To this end, many strategies are tested in preclinical studies such as (i) depleting TAMs using CSF-1R inhibitor [189], (ii) activating TAMs by using agonist anti-CD40 mAbs [148, 190], (iii) reprogramming TAMs to induce pro-inflammatory and antitumor immune response by using TLR agonists [186], (iv) promoting TAMs phagocytic activity by using anti-CD47 mAbs [130, 191] and (v) inhibiting TAMs recruitment by targeting the C-C motif chemokine ligand 2/C-C receptor 2 (CCL2/CCR2) axis [192]. CCL2 chemokine production in GBM TME has also been shown to be essential for the recruitment of MDSCs and Tregs [193]. Blockade of CCL2 is therefore a promising approach to overcome GBM TME immunosuppression [194]. Therapies targeting myeloid cells are even more interesting to use in combination with ICBs knowing that they express high levels of PD-L1. This imply furthermore the great potential of targeting both innate and adaptive immunity for GBM treatment.

In addition, development of technologies that improve immunotherapeutic combination against different targets is necessary. As an example, bispecific antibodies targeting two different antigens has shown to be a valuable approach for combination strategies [195]. Antibodies hold a fundamental place in cancer immunotherapy strategies. However, the BBB limits the passage of most of the large molecules such as mAbs [196]. Many physicochemical properties determine the ability of a compound to pass the BBB (e.g., size, lipophilicity, molecular weight, degree of hydrogen bonding) and the delivery of many drugs across the BBB is consequently insufficient [198]. Therefore, improving delivery systems needs to be considered in order to increase delivery at the tumor site. In this regard, novel cyclic peptides modulating the BBB enhanced the brain delivery of mAbs [196]. Similarly, focused ultrasound-mediated BBB disruption improved anti-CD47 mAbs delivery to GBM tumors [199].

To improve local administration, the use of drug delivery systems enhancing brain penetration for intratumoral administration is highly valuable to improve drug distribution and sustained-release. It was demonstrated that densely PEGylated PLGA-based nanoparticles enhanced the penetration of paclitaxel in the brain tissues and therefore improved the treatment efficacy [200, 201]. Similarly, development of novel implantable biomaterials to improve administration in GBM resection cavity and prevent tumor recurrences are encouraged [202, 203]. Among other technologies, a thermoreversible biodegradable chitosan-based hydrogel containing therapeutic T cells showed encouraging results in GBM, offering an interesting platform for local immunotherapy [204]. Furthermore, nanocarriers have to be developed to enhance the local immune response, turning GBM “cold” tumors into “hot” tumors and therefore promoting the infiltration of immune cells. To do so, inducing ICD of GBM cell by using immunostimulant nanocarriers can facilitate the antitumor immune response and improve therapeutic effects [205, 206]. In addition, new administration routes need to be explored. In this regard, intranasal administration is providing promising results [207, 208]. Finally, scalability and clinical translation must be considered when developing such systems.

## Conclusions

It was long thought that immunotherapy could not be applied to GBM (e.g., because of the BBB, multifactorial immunosuppressive TME, tumor heterogeneity, etc.). The use of ICBs as immunotherapy during the past decade has revolutionized cancer treatments given their ability to improve patient outcomes. However, ICBs for GBM are still ineffective, as proven by the recent phase III clinical trials that did not reach their primary endpoints when administered in monotherapy. Likewise, vaccines added to the SOC did not improve survival.

In contrast, preclinical studies on combination immunotherapy showed encouraging results. The most successful strategies in terms of survival and immune benefits are the ones that impact the cancer-immunity cycle at different time and/or locations, inducing both stimulation of the immune response and inhibition of immunosuppressive components. Combination of ICBs with chemotherapy, radiotherapy inducing ICD or vaccines have been extensively studied and demonstrated significant improvement over monotherapies. Besides, while immunotherapy for cancer is mainly focusing on factors regulating T cell activation, concomitant targeting of both innate and adaptive immunity holds great promise for GBM treatment. The simultaneous use of immunotherapeutic agents targeting different arms of the immune system is starting to be largely tested in GBM clinical trials, given the success of combination immunotherapy in other cancers and in GBM preclinical studies. There are currently nine ongoing clinical trials (phase I or II) studying the combination of multiple ICBs for GBM treatment [209]. The combination of ICBs with radiotherapy or vaccination is also being tested, with twelve and seven ongoing clinical trials, respectively [209].

We believe that currently ongoing clinical trials and preclinical research on combination strategies will provide key information and better survival without major side effects and that immunotherapies will be added to the SOC for patients facing GBM in the near future.

## Data Availability

Not applicable.

## Abbreviations

1-MT: 1-Methyltryptophan
APC: Antigen-presenting cell
BBB: Blood−brain barrier
BCNU: Bis-chloroethyl nitrosurea
CCL2: C-C motif chemokine ligand 2
CCR2: C-C receptor 2
CNS: Central nervous system
CSC: Cancer stem cell
CSF: Colony stimulating factor
CTLA-4: Cytotoxic T-lymphocyte antigen 4
CXCR4: C-X-C chemokine receptor 4
FDA: Food and Drug Administration
GBM: Glioblastoma
GITR: Glucocorticoid-induced tumor necrosis factor-related protein
GM: Granulocyte-macrophage
ICB: Immune checkpoint blockade
ICD: Immunogenic cell death
IDO: Indoleamine 2,3-dioxygenase
IFN: Interferon
LAG-3: Lymphocyte activation gene 3 protein
mAb: Monoclonal antibody
MDSC: Myeloid-derived suppressive cell
MHC: Major histocompatibility complex
NK: Natural killer
PD-1: Programmed cell death-1
PD-L1: Programmed death ligand-1
RT: Radiotherapy
SIRPα: Signal regulatory protein alpha
SOC: Standard of care
SRS: Stereotactic radiosurgery
STING: Stimulator of interferon gene
TAMs: Tumor-associated microglia and macrophages
TCR: T cell receptor
Th1: Type 1 helper
TIGIT: T cell immunoreceptor with Ig and ITIM domains
TIL: Tumor-infiltrating lymphocyte
TIM-3: T cell immunoglobulin and mucin domain containing-3
TLR: Toll-like receptor
TME: Tumor microenvironment
TMZ: Temozolomide
Treg: T regulatory cell
VEGF: Vascular endothelial growth factor
